# Assessing user experience with the Bioline™ HCV point-of-care test in primary healthcare settings: a mixed-methods study

**DOI:** 10.1186/s12913-025-12634-8

**Published:** 2025-04-01

**Authors:** Evans Duah, Evans Mantiri Mathebula, Kuhlula Maluleke, Tinyiko Violet Baloyi, Richard Kobina Dadzie Ephraim, Tivani Mashamba-Thompson

**Affiliations:** 1 Faculty of Health Sciences, School of Health Systems and Public Health, University of Pretoria, Pretoria, 0002 South Africa; 2 Department of Medical Laboratory Science, School of Allied Health Sciences, College of Health and Allied Sciences, University of Cape Coast, PMB, Cape Coast, Ghana

**Keywords:** Acceptability, Primary healthcare, Usability, User-experience, UX

## Abstract

**Background:**

Hepatitis C Virus (HCV) is a major public health challenge, particularly in resource-limited settings with inadequate diagnostic services. The Bioline™ HCV Point-of-Care (POC) test provides a promising solution for improving diagnosis in Primary Healthcare (PHC) clinics without laboratory infrastructure. This study evaluated the test’s usability, acceptability, and deliverability in Ghana using user-oriented REASSURED criteria.

**Methods:**

A convergent parallel mixed-methods design was adopted. Quantitative data was collected through direct observation of Healthcare Workers (HCWs) using audit checklists and analyzed with Stata 16. The analysis included descriptive statistics, inter-rater concordance assessment, and the application of the System Usability Scale (SUS). Qualitative data, analyzed using Atlas.ti 24.2.0, explored user experiences, confidence, storage infrastructure, and suggestions for test design improvement through in-depth interviews.

**Results:**

The quantitative audit included 81 non-laboratory HCWs, with 22 participating in in-depth interviews. The test scored 88.7 on the SUS (95% CI: 86.40-90.88), with 88% of HCWs rating it as easy or very easy to use. Most HCWs (81.5%) successfully completed all testing steps independently, achieving 100% inter-rater concordance, but 83% made errors in at least one step, primarily during pre-testing. Qualitative findings revealed widespread acceptance, confidence, and adaptability despite challenges with storage infrastructure.

**Discussion:**

The Bioline™ HCV POC test demonstrated high usability and acceptance among HCWs in resource-limited settings. Enhancements such as improved packaging, simplified information sheets, refined droppers, and additional components like gloves could further optimize usability. These findings support the Sustainable Development Goal (SDG) 3 by enhancing access to timely HCV diagnosis, contributing to Universal Health Coverage, and strengthening health systems in underserved areas.

**Trial registration:**

This study is part of a diagnostic trial registered in the Pan African Clinical Trial Registry (https://pactr.samrc.ac.za) on 24th October 2024 with trial registration number: PACTR202410837698664.

**Supplementary Information:**

The online version contains supplementary material available at 10.1186/s12913-025-12634-8.

## Introduction

Hepatitis C Virus (HCV) infection, often termed a “silent epidemic”, is a major public health challenge due to its typically asymptomatic nature and delayed diagnosis [1]. Globally, over 130 million individuals are affected by HCV [2], with 58 million living with chronic infection [3], of whom more than 50% are unaware of their status [4]. Sub-Saharan Africa (SSA) accounts for over 2% of the global HCV burden, with more than 200,000 viral hepatitis-related deaths annually [5, 6]. Ghana, like most SSA countries, is impacted by HCV, with studies reporting an HCV seroprevalence of 1–3% [7–9], though national viraemic prevalence remains undocumented.

The World Health Organization (WHO) promotes innovative diagnostic approaches to ensure equitable access and linkage to care, aiming to reduce viral hepatitis mortality by 65% and new infections by 90% by 2030 [10]. While countries like Egypt have expanded community-based HCV testing to meet diagnostic demands [11], Ghana faces challenges in addressing disparities between urban and rural, hard-to-reach communities.

Early detection is critical for linkage to care and treatment with Direct-Acting Antivirals (DAAs) [3]. Point-of-Care (POC) diagnostics offer near-bedside rapid testing, addressing diagnostic needs in hard-to-reach, resource-limited communities typical of low-middle-income countries (LMICs) [12]. In Ghana, there is a significant demand for POC services in Primary Healthcare (PHC) clinics, especially in remote areas where centralized laboratories are either unavailable or located hours away [13]. The Bioline™ HCV POC test (Abbott Rapid Diagnostics) provides an accessible alternative, promoting equitable access to diagnostics and facilitating early detection [14]. This test is WHO prequalified, market-authorized, and CE-marked for use in multiple regions [14].

However, limited research exists on Healthcare Workers’ (HCWs) User Experience (UX) with HCV POC tests in SSA’s PHC settings. Most diagnostic trials in SSA focus on clinical performance in laboratory environments [15], often excluding African populations and favoring high-income countries. Meanwhile, user-oriented indicators, such as ease of specimen collection, user-friendliness, rapidity, and deliverability, are commonly assessed using HCV self-tests [16–18].

The present study constituted part of a diagnostic trial that aimed to evaluate the Bioline™ HCV POC test in PHC settings in Ghana using the Real-time connectivity, Ease of specimen collection, Affordability, Sensitivity, Specificity, User-friendliness, Rapidity and robustness, Equipment-free, and Deliverability (REASSURED) criteria as a benchmark [19]. Guided by the Morville UX Honeycomb model [20], we specifically investigated the UX, employing a mixed-methods design to evaluate the usability, acceptability, and deliverability of the Bioline™ HCV POC test among non-laboratory HCWs in PHC clinics in Ghana’s Central Region.

## Methods

### Study design

A convergent parallel mixed-methods design was used to evaluate the UX of the Bioline™ HCV POC test as part of a broader diagnostic trial registered in the Pan African Clinical Trial Registry (PACTR202410837698664) whose protocol is published elsewhere [19]. The study was conducted from August-December 2024.

### Study setting

**Fig. 1:**
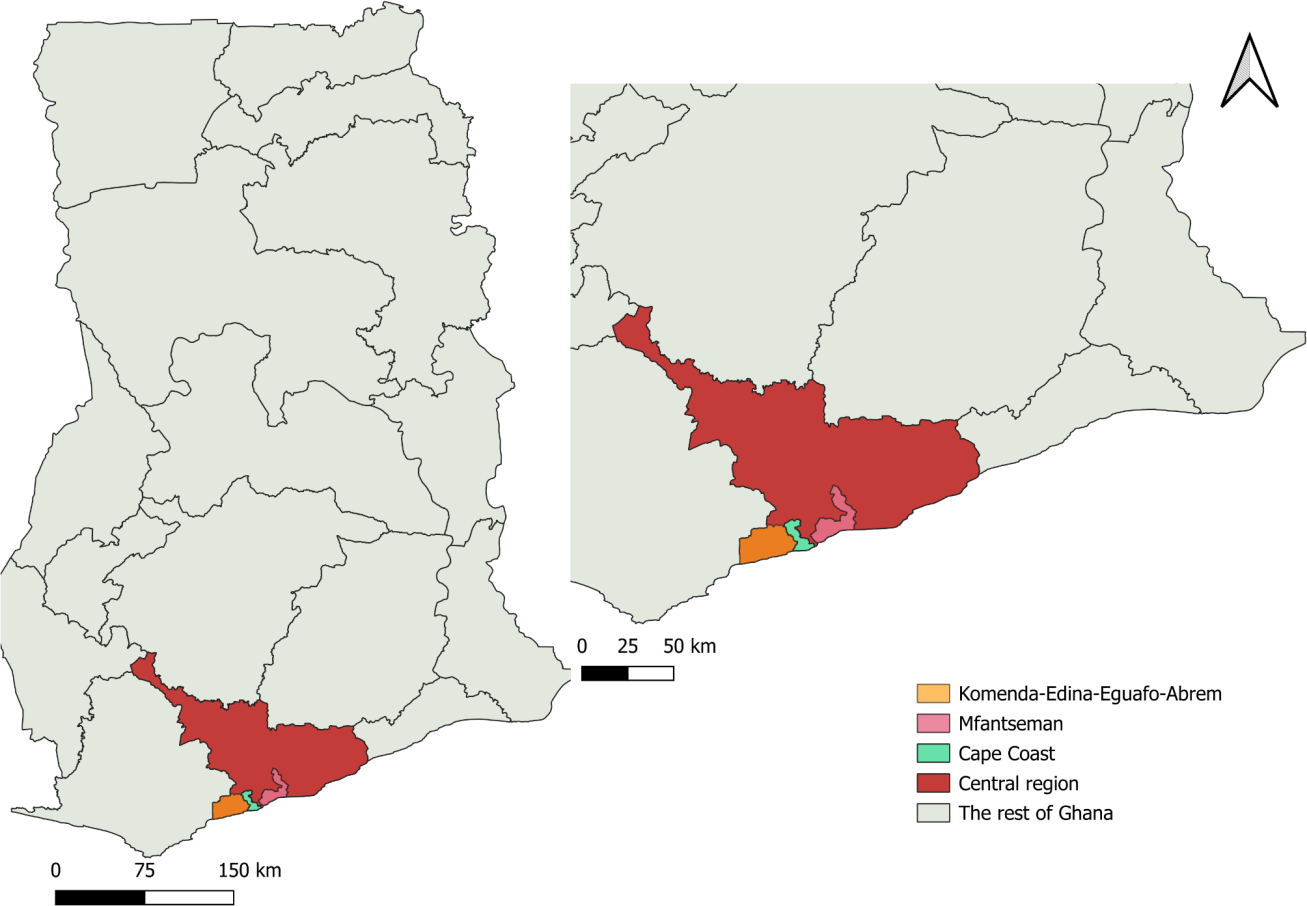
Map of the Central Region of Ghana highlighting the three study districts

The study was conducted in the Central region of Ghana (5.6444° N, 1.2891° W) (Fig. 1), covering 42 Community-based Health Planning and Services (CHPS) facilities across Cape Coast, Komenda-Edina-Eguafo-Abirem (KEEA), and Mfantseman districts. These facilities, identified as PHC clinics, were purposively selected based on the study objectives (Additional file 1).

### Study population

Non-laboratory HCWs at the PHC clinics including nurses and midwives were recruited to participate in this study.

### Participants selection and eligibility criteria

#### Inclusion criteria

- All PHC clinics within the three districts (Cape Coast, KEEA, and Mfantseman) were eligible to be included in the study.
- All staff of the PHC clinics were eligible to participate in the study.

#### Exclusion criteria

- PHC clinics that had laboratories were excluded from the study.
- All PHC clinics that did not have physical structures were excluded from the study.
- All laboratory staff of the clinics were excluded from the study.
- Participants who withdrew consent were excluded from the study.
- Interns and non-medical support staff at the PHC clinics were excluded from the study.

HCWs who met the eligibility criteria were selected through purposive sampling.

#### Sample size

Using Yamane’s sample size formula, a minimum of 80 HCWs were required to obtain a representative sample from the available 100 HCWs at the PHC clinics to ensure study validity [21].

$$\:{n}_{1}\ge\:N/(1+N{e}^{2})$$

Where:

N = The total number of HCWs available at the PHC clinics in the three districts = 100

e = margin of error = 5% =0.05 at 95% Confidence Interval

$$\:n_1\geq\:N/(1+Ne^2)$$

$$\:n_1\geq\:100/((1+100\left(0.05\right)^2)$$

$$\:{n}_{1}\ge\:80$$

Also, the minimum required sample size for the qualitative component was set at 20 in-depth interviews until data saturation was reached.

### Study procedures

The Bioline™ HCV test is a manually performed, immunochromatographic IVD for detecting HCV antibodies in human serum, plasma, or whole blood at POC within 5 to 20 min [14]. It has a sensitivity of 99.3% and specificity of 98.1% [14]. The test is WHO prequalified, CE marked [14], and registered with the Ghana FDA (FDA/D.21–11702) [19]. Its packaging includes test cassettes, safety lancets, specimen droppers, alcohol swabs, diluent, and instructions (Additional file 2), designed for resource-limited settings, especially in SSA.

The manufacturer’s test instructions included both text and diagramatic illustrations in multiple languages. The Bioline™ HCV test workflow adapted from the test instruction leaflet for this study involved a 23-step process categorized into 3 phases i.e. pre-testing, testing and post-testing (Additional file 3).

#### Quantitative methods

The principal investigator and trained research assistants (medical laboratory scientists, nurses, and public health officers) demonstrated the Bioline™ HCV test workflow to participants according to the manufacturer’s instructions. A baseline questionnaire collected sociodemographic data, including sex assigned at birth, education, occupation, and rank. Participants were paired to perform the HCV test on each other, interpreting results while referring to the instructions. Research assistants observed the 23-step process, documenting errors and difficulties (Additional file 3).

Inter-reader and inter-operator concordance were estimated by comparing participants’ results with those of the research assistants. Participants also tested one of randomly-selected five blinded standard samples (A to E), and the results were compared to known outcomes. A 10-item Likert-scale questionnaire was used to assess the participants’ experiences after testing (Additional file 3).

Finally, the standard and validated System Usability Scale (SUS) was employed to evaluate user interactions with the test and their experiences [22]. The standard SUS, comprising ten 5-point Likert-scale items alternating between positive (odd) and negative (even) statements, assesses test usability and overall user satisfaction (Additional file 3). The SUS scores range from 0 to 100, with scores ≤ 51.8 indicating the system requires fixing; 51.9–67.9: system improvement is recommended; 68–73.9: high usability but potential for improvement exists; 74–80.2: high usability; ≥80.3: excellent usability [23]. The standard SUS score for a set of raw item ratings from each respondent was calculated using the following formula:

$$\:SUS\:score=\left(\sum\limits_{i=\text{1,3},\text{5,7},9}{(score}_{i}-1)+\sum\limits_{i=\text{2,4},\text{6,8},10}{(5-score}_{i})\right)\times\:\:2.5$$

Statements 1, 3, 5, 7, and 9 represent the odd or positive statements, while statements 2, 4, 6, 8, and 10 represent the even or negative statements. The frequencies of responses for each Likert scale option (1–5) for each statement were enumerated and adjusted according to the following adjusted score rules:

- Odd-numbered items: Adjusted score = Response score– 1 = 0, 1, 2, 3, 4
- Even-numbered items: Adjusted score = 5 - response score = 4, 3, 2, 1, 0

To obtain total adjusted scores, the frequency of each response was multiplied by its adjusted score. The cumulative adjusted score was calculated as the sum of the total adjusted scores. The average SUS for N number of respondents was computed as:

$$\:Average\:SUS\:score=\frac{SUS\:score}{N}$$

#### Qualitative methods

Concurrently, the study explored users’ experiences and acceptability of the Bioline™ HCV test through qualitative methods. In-depth interviews were conducted using a semi-structured guide (Additional file 4), consisting of 15 open-ended questions about participants’ experiences, confidence, usability, reliability of the test instructions, challenges, ease of sample collection, quality assurance, and recommendations. Interviews, lasting 20–45 min, were conducted in Fanti, Twi, or English, based on participant preference. They were audio-recorded, with field notes taken to supplement the recordings.

### Data management and analysis

#### Quantitative data

Quantitative data were entered into Microsoft Excel, cleaned for duplicates, missing data, and errors, then exported to Stata/IC version 16 [24]. Descriptive statistics were used to summarize the study population, presented as frequencies and percentages. Estimates of errors, difficulties, and assistance during the hands-on audit were generated. HCV invalid ratings by PHC non-laboratory HCWs were also estimated. Inter-rater concordance (inter-reader and inter-operator) was calculated as percentage agreement and Cohen’s kappa coefficient. SUS scores were computed using the provided formula. Statistical results were presented in tables and graphs, with all tests two-sided, a 95% confidence interval, and *p* < 0.05.

#### Qualitative data

Interview recordings were transcribed verbatim in Microsoft Word, with translations of local languages into English, and then exported to ATLAS.ti software version 24.2.0 [25]. Two independent coders (ED and TVB) used thematic analysis to inductively code the data in parallel [26]. Inter-coder reliability was assessed using percent agreement (85.4%), the Holsti index (0.91), and Krippendorff’s alpha-binary coefficient (0.909), all indicating acceptable agreement (≥ 80% or ≥ 0.80) (Additional file 5) [27]. Major themes and sub-themes were generated to explore users’ experiences of the Bioline™ HCV test, following Braun & Clarke’s six-phase framework: familiarizing with data, extracting codes, generating themes, reviewing themes, defining themes, and writing up [26]. Results were presented as relevant quotes from the transcripts.

## Results

### Characteristics of the study participants

Table 1 presents the sociodemographic characteristics of 81 non-laboratory HCWs with a median age of 34 years (IQR:31–37), recruited from PHC clinics across three districts: Cape Coast (34.6%), Mfantseman (33.3%), and KEEA (32.1%). Participants had a median total work experience of 8 years (IQR:4–11). Most participants were females (91.4%), with certificate-level education (59.3%) and diplomas (33.3%). Nurses constituted the largest occupational group (76.6%), followed by midwives (22.2%). Participants included community health nurses (20.9%) and senior community health nurses (23.5%) being the most common. Leadership roles were held by 39.5% of participants, with 34.6% serving as general managers of the PHC clinic.

**Table 1:** Sociodemographic characteristics of study participants

Variables	*N* = 81 *n*(%)
Median age, years (IQR^*^)	34(31–37)
Median total working experience, years (IQR^*^)	8(4–11)
Median working experience in that specific PHC clinic, years (IQR^*^)	2(1–4)
Sex assigned at birth	
Male	7(8.6)
Female	74(91.4)
Highest level of education	
Certificate (1–2 years)	48(59.3)
Diploma (3 years)	27(33.3)
Degree (4 years)	6(7.4)
Occupation	
Health Assistant	1(1.2)
Midwife	18(22.2)
Nurse	62(76.6)
Rank	
Community health nurse	17(20.9)
Senior community health nurse	19(23.5)
Principal community health nurse	11(13.6)
Enrolled nurse	3(3.7)
Senior enrolled nurse	5(6.2)
Principal enrolled nurse	1(1.2)
Midwifery officer	7(8.7)
Senior midwifery officer	2(2.5)
Nursing officer	1(1.2)
Principal health assistant	1(1.2)
Senior public health nurse	1(1.2)
Staff midwife	2(2.5)
Senior staff midwife	7(8.7)
Staff nurse	3(3.7)
Senior staff nurse	1(1.2)
Leadership role	
General manager	28(34.6)
RCH^#^ unit manager	4(4.9)
None	49(60.5)
Distribution by district	
KEEA^∞^	26(32.1)
Cape Coast	28(34.6)
Mfantseman	27(33.3)

^*^IQR Inter-quartile range, *PHC* Primary healthcare

^#^*RCH* Reproductive and child health

^∞^*KEEA* Komenda-Edina-Eguafo-Abrem

The in-depth interviews included 22 HCWs, of whom 17 were facility managers. Detailed description is provided in Additional file 6.

Table 2 summarizes the characteristics of the PHC clinics included in the study. These were 42 CHPS compounds with physical structures distributed across three districts: KEEA (35.7%), Cape Coast (31.0%), and Mfantseman (33.3%). The condition of roads leading to the clinics was predominantly poor but pliable (59.5%). The majority of the clinics (95.2%) lacked air-conditioned storage infrastructure.

**Table 2:** Characteristics of the PHC clinics

Variables	*N* = 42 *n*(%)
District	
KEEA^*^	15(35.7)
Cape Coast	13(31.0)
Mfantseman	14(33.3)
Condition of road leading to PHC^#^ clinic	
Good	15(35.7)
Poor but pliable	25(59.5)
Non-pliable	2(4.8)
Availability of air-conditioned storage room	
No	40(95.2)
Yes	2(4.8)

^*^KEEA Komenda-Edina-Eguafo-Abrem

^#^*PHC* Primary healthcare

Results of the hands-on usability audit of the Bioline™ HCV POC are presented in Table 3. Out of the 81 study participants, 33 opted to perform self-testing, either because they were the only healthcare worker on duty at the time of the study or to maintain confidentiality, as they did not want a colleague (testing partner) to know their HCV status. Overall, errors were observed in about 83% of the participnats for at least one step, common among those with testing partners (100% vs. 57.6%, *p* < 0.0001). Pre-testing errors were common, with 64.2% failing to wash and dry their hands, 62.9% not reading or using the test information sheet, and 60.5% not wearing examination gloves. Specifically, significantly higher percentage of self-testers failed to wear examination gloves (100% vs. 33.3%, *p* < 0.0001) compared with those with testing partners. However, most participants with testing partners failed to read or use the test information sheet (72.9% vs. 48.5%, *p* = 0.03) compared with self-testers. During testing, 37.0% failed to wipe away the first drop of blood, and 27.2% did not collect the required 10 µl of blood. There were minimal or no post-testing errors. Difficulties were noted in 18.5% of participants for at least one step, primarily in removing the test device from the foil (13.6%) and using the specimen dropper (8.6%). Assistance was provided in 18.5% of cases for at least one step, mainly for timing the test (12.4%). Despite the errors and challenges, 81.5% completed all steps without assistance and reported correct test results, with self-testers showing a higher success rate than testing partners (87.9% vs. 77.1%).

**Table 3:** Bioline™ HCV POC hands-on usability audit: observed errors, difficulties and assistance provided

Variables	Overall *N* = 81	Testing partner *N* = 48	Self-test *N* = 33	*P*-value
	*n*(%)	*n*(%)	*n*(%)	
**Observed errors**				
*Pre-testing*				
Failed to wash hands and dried	52(64.2)	29(60.4)	23(69.7)	0.39
Replaced handwash with hand sanitizer	23(28.4)	9(18.8)	14(42.4)	-
Didn’t read/use test information sheet	51(62.9)	35(72.9)	16(48.5)	0.03*
Didn’t place the test kit on a flat surface	1(1.2)	1(2.1)	0(0.0)	1.00
Didn’t wear examination gloves	49(60.5)	16(33.3)	33(100.0)	< 0.0001*
Failed to label the test kit	28(34.6)	19(39.6)	9(27.3)	0.25
*Testing*				
Chose the wrong finger for fingerpricking	0(0.0)	0(0.0)	0(0.0)	-
Didn’t massage and warm finger	5(6.2)	4(8.3)	1(3.0)	0.64
Failed to clean the finger with an alcohol swab and let it dry	0(0.0)	0(0.0)	0(0.0)	-
Didn’t press down firmly to prick their skin	1(1.2)	1(2.1)	0(0.0)	1.00
Unsafe disposal of the used lancet	0(0.0)	0(0.0)	0(0.0)	-
Didn’t wipe away the first drop of blood	30(37.0)	19(39.6)	11(33.3)	0.57
Didn’t use the specimen dropper	1(1.2)	1(2.1)	0(0.0)	-
Failed to collect the blood up to the marked ring of the specimen dropper (10 µl)	22(27.2)	15(31.3)	7(21.2)	0.32
Didn’t drop the whole blood into the round specimen well (marked ‘S’)	1(1.2)	0(0.0)	1(3.0)	0.65
Unsafe disposal of used specimen dropper	0(0.0)	0(0.0)	0(0.0)	-
Didn’t use the assay diluent	0(0.0)	0(0.0)	0(0.0)	-
Failed to dispense exactly 4 drops of the assay diluent	8(9.9)	7(14.6)	1(3.0)	0.13
Didn’t close the cap of the assay diluent after use	1(1.2)	1(2.1)	0(0.0)	1.00
Didn’t time the test	10(12.4)	8(16.7)	2(6.1)	0.17
*Post-testing*				
Read the test results outside the stipulated time (5–20 min)	0(0.0)	0(0.0)	0(0.0)	-
Read/interpreted the results incorrectly	0(0.0)	0(0.0)	0(0.0)	-
Unsafe disposal of the used test kit	0(0.0)	0(0.0)	0(0.0)	-
Errors observed for at least one step	67(82.7)	48(100.0)	19(57.6)	< 0.0001*
**Observed difficulties**				
Removing the test device from the foil	11(13.6)	6(12.5)	5(15.2)	0.73
Using the specimen dropper	7(8.6)	7(14.6)	0(0.0)	0.41
Experienced difficulties for at least one step	15(18.5)	12(25.0)	3(9.1)	0.09
**Assistance provided**				
Asked for more information mid-way testing	1(1.2)	1(2.1)	0(0.0)	1.00
Reminded by testing partner to clean finder with alcohol swab	1(1.2)	1(2.1)	0(0.0)	1.00
Collecting blood with specimen dropper	4(4.9)	2(4.2)	2(6.1)	0.41
Dispensing blood from the specimen dropper unto the test kit	1(1.2)	0(0.0)	1(3.0)	1.00
Timing	10(12.4)	8(16.7)	2(6.1)	0.17
Assistance provided for at least one step	15(18.5)	11(22.9)	4(12.1)	0.26
All steps completed correctly without assistance and test results reported correctly	66(81.5)	37(77.1)	29(87.9)	0.26

^*^Statistically significant at *p* < 0.05

The qualitative findings highlight similar challenges as part of the five emerging themes contributing to these difficulties and errors, particularly issues with the test instruction sheet and specimen dropper (Additional file 7).

#### Challenge with reading/using the test instruction sheet

Participants found the instruction leaflet cumbersome due to its size, multiple languages, small font, and overwhelming appearance (Additional file 8).

*“When I saw the leaflet*,* I got scared. I was like*,* “Ei*,* what is this big thing? When will I even finish reading this? (She laughed)”* (HCW of PHC clinic 19)

*“I had a little difficulty with the pamphlet (instruction sheet). It’s quite large and includes multiple languages*,* which made it hard to locate the specific information needed to perform the test”* (HCW of PHC clinic 11)

“*The font is too small for some of us with poor eyesight”* (HCW of PHC clinic 13)

#### Challenge with using the specimen dropper

Approximately half of the participants struggled with the sample dropper (Additional file 8).

*“I had a problem with the pipette. I had to press it before picking up the sample. It requires some skill to do that. If you don’t know how*,* you can’t take enough sample for the test”* (HCW of PHC clinic 14)

However, the study participants suggested several modifications to enhance the usability and functionality of the Bioline™ HCV POC test (Additional file 7).

#### Suggestion to modify the test package

Participants emphasized the need for integrating all test components into a single packaging unit to prevent shortages and improve convenience.

*“Okay. I would suggest they improve the packaging. If the pipette (dropper) and lancet are included in the foil with the cassette*,* it would be more convenient. That way*,* when you pick one*,* everything you need is inside. Currently*,* since the pipettes and lancets are packed separately*,* sometimes we run out of them even though we still have cassettes available. Including them together would solve this issue”* (HCW of PHC clinic 1)

#### Suggestion to include additional consumables

Recommendations were made for manufacturers to consider.

*“They should include additional droppers since we may accidentally drop them while working”* (HCW of PHC clinic 2)

***“****Adding lancet holders would be very helpful*,* as it would reduce clients’ fear of needles by hiding the needle during finger pricks…* (HCW of PHC clinic 3)

*“You should add a lancet holder to automate the pricking process and protect us from needle pricks during our work. This would also reduce the pain caused by the prick”* (HCW of PHC clinic 4)

*“We often lack sufficient gloves. Including gloves*,* even simple rubber gloves*,* in the test package would greatly enhance safety”* (HCW of PHC clinic 7)

*“…including a timer would be beneficial to help with timing*,* as we might forget to check the result on time”* (HCW of PHC clinic 3)

#### Suggestion to modify specimen dropper

Similarly, recommendations were made for the modification of the sample dropper.

*“I would recommend changing the pipette (dropper). It’s difficult to pick the sample with it compared to what we are used to with the malaria RDT. With the malaria RDT*,* you don’t need to press or hold anything to pick the sample. It picks the sample with just a touch”* (HCW of PHC clinic 6)

#### Suggestion to modify test instruction sheet

Participants made suggestions for test design improvement.

*“…the leaflet should be simplified. I don’t know if this is in Chinese*,* but if you’re bringing the test to this level of healthcare in Ghana*,* most of us are certificate and diploma holders. When someone sees a language other than English*,* they might not even continue to look at the instructions. So*,* I would recommend designing a country-specific leaflet with test instructions in diagrams showing the steps*,* to make it simpler and easier to read”* (HCW of PHC clinic 14)

**Fig. 2:**
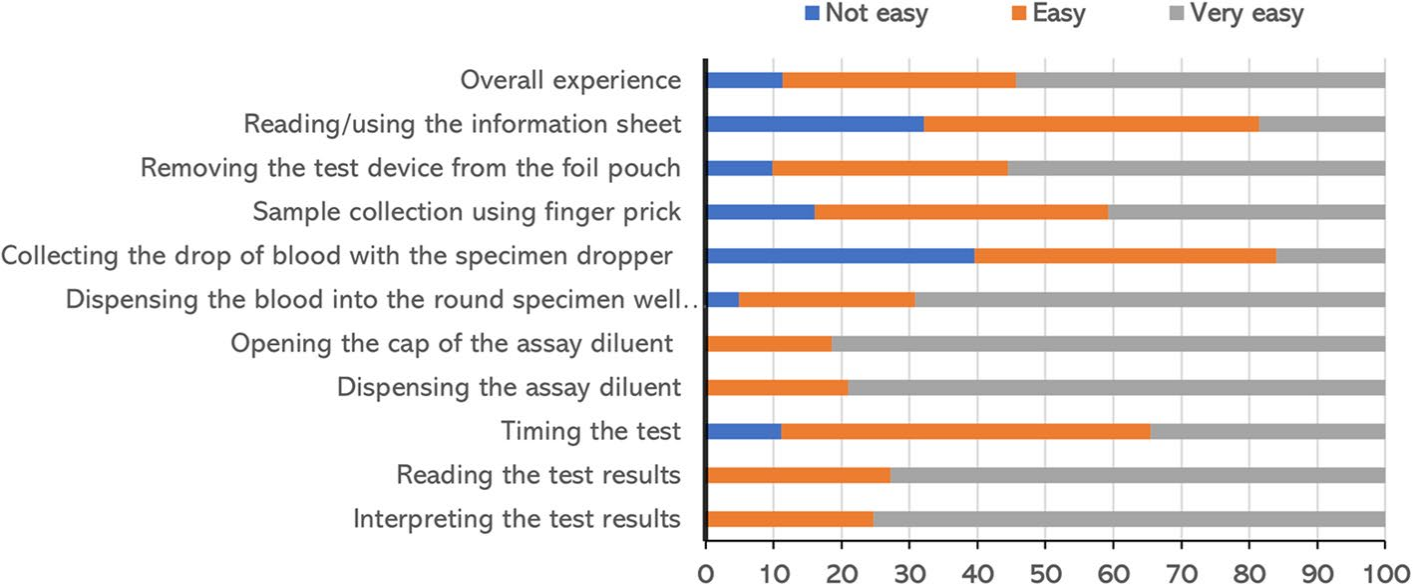
Ease-of-use of the Bioline™ HCV POC test: user-experience audit

The HCWs shared their experiences on the ease-of-use of the Bioline™ HCV POC test as illustrated in Fig. 2. Overall, 88% of the HCWs found the test easy or very easy to use with various ease-of-use ratings in the testing steps. The commonest difficulty was with the use of the sample dropper (Fig. 2).

The in-depth interviews complement the UX findings, highlighting the test’s ease-of-use and the participants willingness to use and recommend to other PHC clinics outside the study districts (Additional file 7).

#### Ease-of-use of the test

The Bioline™ HCV POC test was widely regarded as simple and user-friendly by the HCWs.

*“Every equipment needed for the test is found in the small box. You don’t need any other equipment to perform the test*,* which makes it very simple to use”* (HCW of PHC clinic 18)

*“The test kit is quite compact and lightweight. It’s something we can easily work with”* (HCW of PHC clinic 11)

#### Willingness to use test in the PHC clinics

As the first point of contact for healthcare in underserved areas, the partcipants expressed strong willingness to use the test.

*“Yes. As the first point of contact in the hierarchy of Ghana’s health service*,* having this test kit will make our work much easier. Clients will no longer need to travel long distances and incur financial costs for laboratory tests” (HCW of PHC clinic 7)*

#### Willingness to recommend to other PHC clinics

Participants noted that misconceptions about higher healthcare levels often deter community members from seeking care, making PHC clinics the preferred point for diagnostic services hence their willingness to recommend to all PHC settings.

*“Yes. Most people in these communities see hospitals as something big*,* so they are scared to go there. They are comfortable coming to the CHPS compounds since we are closer to their homes. It will also make the HCV diagnostic service easily accessible to them” (HCW of PHC clinic 15)*

*“Yes*,* it will be very helpful because most community members prefer accessing healthcare in CHPS zones (PHC clinics). Even when referred to a higher level of care*,* they often refuse to go. They are scared of larger health facilities. Having this test will help us diagnose them here and provide appropriate follow-up treatment” (HCW of PHC clinic 12)*

**Fig. 3:**
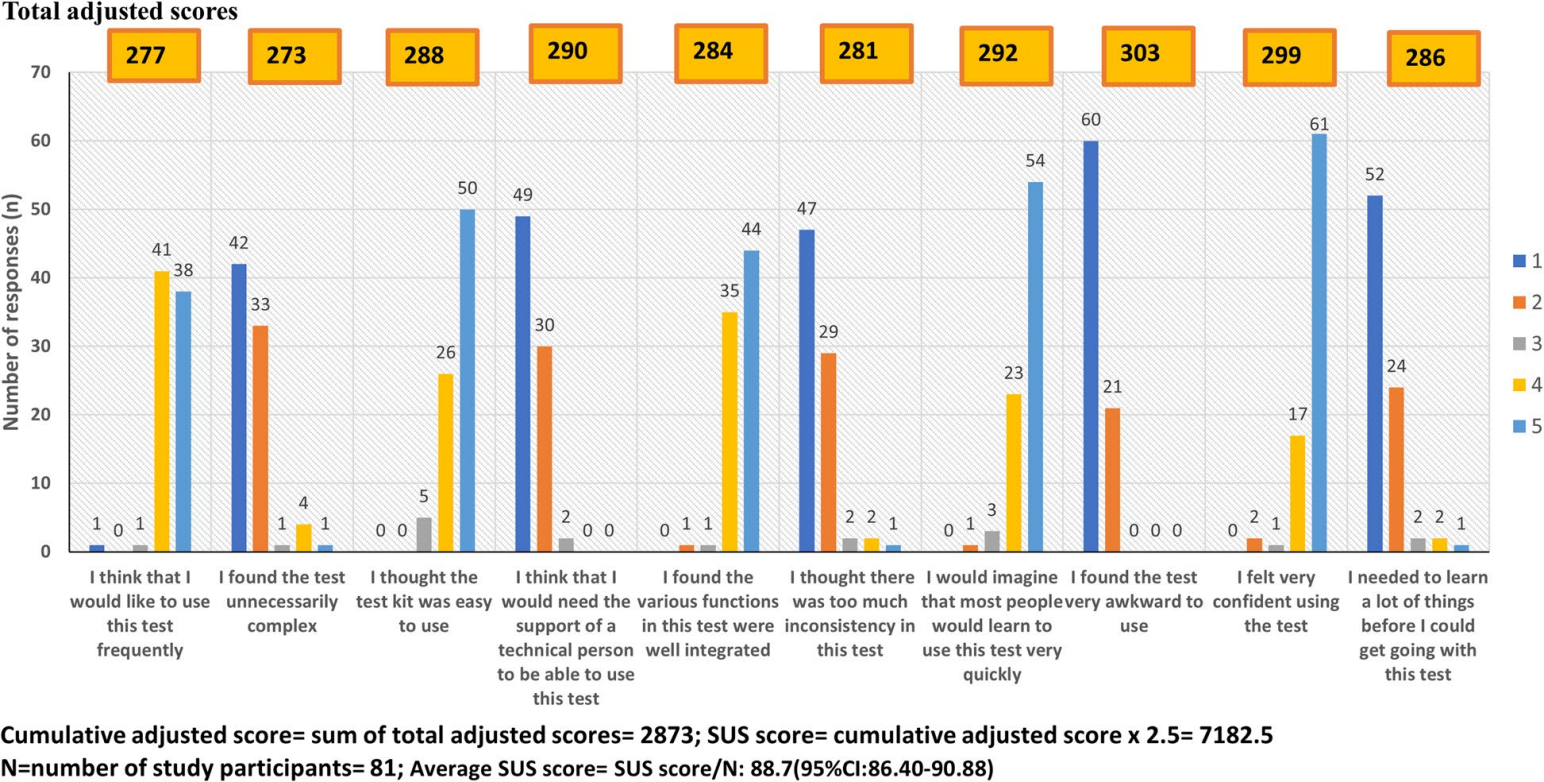
Bioline™ HCV POC test usability assessment using the SUS scale

The overall usability score for the Bioline™ HCV POC was 88.7 (95% CI: 86.40–90.88) based on the validated SUS scale as shown in Fig. 3.

The overall inter-reader and inter-operator concordance was high using both the person-testing and standard sample testing approaches (100%, Cohen’s kappa coefficient:1.00 ± 0.11, *p*-value < 0.0001), with no invalid rating recorded (Additional file 9).

## Discussion

The overall usability score of the Bioline™ HCV POC test was 88.7 (95% CI: 86.40–90.88), an excellent rating according to the SUS [22]. This finding highlights the test’s ease of use, acceptability, and practicality, making it suitable for adoption in resource-limited PHC settings with minimal additional resources or training. While the SUS is widely used in technology and consumer electronics [22], its application in healthcare and diagnostics is growing. For instance, similar usability scores were recorded for the SureStatus POC SARS-CoV-2 Antigen test (85.0) in India and Germany [28], and the Abbott i-STAT^®^ POC blood analyzer (84.0) in Canada [23]. Comparable studies in South Africa also reported high usability scores (90.7–94.9%) for HCV self-tests [29].

In this study, 88% of HCWs found the test easy or very easy to use, consistent with the high usability scores and qualitative feedback. The compact design, rapid results, and familiarity with rapid diagnostic testing contributed to this positive perception. The finger-prick procedure was also straightforward, with minimal discomfort for patients. Familiarity with dual-format instruction sheets further improved ease of use, reflecting findings from other studies where usability scores were similarly high among lay users of HCV self-tests in South Africa [29], and Georgia [16].

Approximately 81.5% of HCWs successfully completed all test steps without assistance and reported accurate results, with no invalid ratings, yielding 100% inter-rater reliability. This performance surpasses findings from a similar study in Georgia, where 60–80% of lay users correctly completed HCV tests without assistance [16]. This discrepancy may reflect differences in target populations, with HCWs being more familiar with diagnostic procedures than lay users. This was evident in lay users occasionally misinterpreting the test lines and facing difficulties distinguishing between positive and negative results [16].

Despite these successes, errors were observed in 83% of participants during at least one testing step, primarily in the pre-testing phase. Common errors included failure to wash hands (64.2%), not using the test information sheet (62.9%), and neglecting to wear gloves (60.5%). These lapses could be attributed to overconfidence and routine familiarity with the procedures, which seemed habitual and normalized within PHC settings, as highlighted in the qualitative analysis. Furthermore, many HCWs trusted their testing partners, perceiving little need to wear gloves. The shortage of consumables, such as gloves, reported in most facilities further exacerbates these unsafe practices. In contrast, other studies rather reported post-analytic errors, such as misinterpreting test results [16, 17].

Acceptance of the Bioline™ HCV POC test was high among HCWs, who expressed willingness to recommend and use the test in PHC settings. HCWs emphasized the importance of community-based testing, citing reliance by farming and fishing communities and reluctance to visit higher-level facilities due to financial and cultural barriers. Similar acceptability trends were noted in Kenya, Egypt, Vietnam, and China, where HCV self-tests gained widespread approval despite limited literature on Bioline™ HCV POC testing among HCWs [18].

Deliverability, encompassing physical accessibility and storage capabilities, is critical for deploying the test in hard-to-reach areas. Most PHC clinics in this study had poor infrastructure and lacked temperature-controlled storage. However, HCWs demonstrated adaptability by creating suitable storage environments using existing resources. On the other hand, limited awareness of HCV RDTs among HCWs raised concerns about broader population knowledge in rural areas. For instance, a study in India found that over 50% of at-risk individuals were unaware of HCV testing due to a lack of awareness [30].

This study’s findings have significant implications for scaling up the Bioline™ HCV POC test in resource-limited settings. High usability and acceptability suggest its potential as a viable diagnostic tool, even in infrastructure-poor environments. Recommendations for improving usability include enhancing packaging, instruction sheets, and sample droppers, as well as providing supplementary components like gloves. Targeted training programs are necessary to address knowledge gaps and improve familiarity with HCV RDTs, which could enhance early detection and management of the disease.

Policy efforts should prioritize integrating user-friendly POC tools into national health programs and addressing infrastructural challenges. Continuous implementation research is needed to monitor usability under real-world conditions, optimize delivery mechanisms, and compare performance with alternative diagnostics. The adaptability and resilience demonstrated by HCWs in overcoming logistical challenges underscore the potential for deploying similar tests in other low-resource settings, contributing to global health goals for HCV care and prevention.

Despite its strengths, the study is limited by potential response bias, as HCWs were aware of being observed. However, the use of validated tools like the SUS, well-trained research assistants, and observational audits helped mitigate this risk. The congruence between quantitative and qualitative findings further supports the study’s validity and reliability.

## Conclusion

Our study evaluated the UX of non-laboratory HCWs with the Bioline™ HCV POC test in Ghana, highlighting its usability, acceptability, and deliverability. Despite some procedural errors and infrastructure challenges, HCWs found the test user-friendly, rapid, and suitable for resource-limited settings. The findings suggest the test’s potential for integration, with recommendations for targeted training and design improvements. These outcomes align with SDG 3 by promoting equitable access to HCV diagnostics, advancing Universal Health Coverage, and addressing health disparities in underserved regions.

## Supplementary Information

Additional file 1.

Additional file 2.

Additional file 3.

Additional file 4.

Additional file 5.

Additional file 6.

Additional file 7.

Additional file 8.

Additional file 9.

## Data Availability

All study materials have been uploaded as supplementary information. All data generated in the study will be made available on request through the corresponding author: evans.duah@tuks.co.za.

## Abbreviations

HCV: Hepatitis C Virus
POC: Point-of-care
PHC: Primary healthcare
HCW: Healthcare worker
WHO: World Health Organization
RDT: Rapid diagnostic test
CHPS: Community-based Health Planning and Services
SUS: System Usability Scale
REASSURED: Real-time connectivity, Ease of specimen collection, Affordablility, Sensitivity, Specificity, User-friendliness, Rapidity and robustness, Equipment-free, and Deliverability
HCVST: HCV self-test
PWID: People who inject drugs
MSM: Men who have sex with men
TG: Transgender
SSA: Sub-Saharan Africa
LMIC: Low- and middle-income country
IVD: In vitro diagnostic
UX: User experience
SDG: Sustainable Development Goals
UHC: Universal Health Coverage

## References

[1] Kretzer IF, Livramento AD, Cunha JD, Gonçalves S, Tosin I, Spada C, Treitinger A, Hepatitis C. Worldwide and in Brazil: silent Epidemic—Data on disease including incidence, transmission, prevention, and treatment. Sci World J. 2014;2014:1–10. 10.1155/2014/827849 PMC4070442 25013871

[2] Moosavy SH, Davoodian P, Nazarnezhad MA, Nejatizaheh A, Eftekhar E, Mahboobi H. Epidemiology, transmission, diagnosis, and outcome of hepatitis C virus infection. Electron Physician. 2017;9(10):5646–56. 29238510 10.19082/5646 PMC5718874

[3] WHO. Hepatitis C. World Health Organization. 2023. [cited 2023 Sep 2]. Available from: https://www.who.int/news-room/fact-sheets/detail/hepatitis-c.

[4] Kowdley KV. Identification of people infected with hepatitis C virus who have never been diagnosed. Gastroenterol Hepatol (N Y). 2019;15(12):669–71. 31892913 PMC6935023

[5] WHO. Hepatitis: disease burden. World Health Oganisation. 2021. Available from: https://www.afro.who.int/health-topics/hepatitis.

[6] Karoney MJ, Siika AM. Hepatitis C virus (HCV) infection in Africa: a review. Pan Afr Med J. 2013;14:44. 23560127 10.11604/pamj.2013.14.44.2199 PMC3612901

[7] Agyeman AA, Ofori-asenso R, Mprah A, Ashiagbor G. Epidemiology of hepatitis C virus in Ghana: a systematic review and meta-analysis. BMC Infect Dis. 2016;16(1):391. 10.1186/s12879-016-1708-7 PMC4977883 27507267

[8] Birjandi MM, Oroei M. The prevalence of positive rapid diagnostic test of hepatitis C virus infection in Ghana. Pan Afr Med J. 2020;36(1):322. 10.11604/pamj.2020.36.322.22490 PMC7603834 33193976

[9] Tachi K. Hepatitis C virus infection in Ghana: time for action is now. Ghana Med J. 2018;52(1):1–2. 30013253 10.4314/gmj.v52i1.1 PMC6026945

[10] WHO. Hepatitis C. World Health Organization, Geneva. 2022. [cited 2022 Jun 10]. Available from: https://www.who.int/news-room/fact-sheets/detail/hepatitis-c.

[11] Hassanin A, Kamel S, Waked I, Fort M. Egypt’s ambitious strategy to eliminate hepatitis C virus: A case study. Global Health: Sci Pract. 2021;9(1):187–200. 10.9745/GHSP-D-20-00234 PMC8087425 33795369

[12] Larkins MC, Thombare A. Point-of-Care Testing. In: StatPearls. Treasure Island: StatPearls Publishing; 2023 [cited 2023 Sep 2]. Available from: http://www.ncbi.nlm.nih.gov/books/NBK592387/.

[13] Heidt B, Siqueira WF, Eersels K, Diliën H, van Grinsven B, Fujiwara RT, Cleij TJ. Point of care diagnostics in Resource-Limited settings: A review of the present and future of poc in its most needed environment. Biosens (Basel). 2020;10(10):133. 10.3390/bios10100133 PMC7598644 32987809

[14] Abbott. Bioline^™^ HCV. Abbott Laboratories. 2023. [cited 2023 Sep 2]. Available from: https://www.globalpointofcare.abbott/ww/en/product-details/bioline-hcv.html.

[15] Duah E, Mathebula EM, Mashamba-Thompson T. Quality assurance for hepatitis C virus Point-of-Care diagnostics in Sub-Saharan Africa. Diagnostics. 2023;13(4):684. 36832172 10.3390/diagnostics13040684 PMC9955859

[16] Fajardo E, Watson V, Kumwenda M, Usharidze D, Gogochashvili S, Kakhaberi D, Giguashvili A, Johnson CC, Jamil MS, Dacombe R, Stvilia K, Easterbrook P, Ivanova Reipold E. Usability and acceptability of oral-based HCV self-testing among key populations: a mixed-methods evaluation in Tbilisi, Georgia. BMC Infect Dis. 2022;22(1):510. 35641908 10.1186/s12879-022-07484-2 PMC9154030

[17] Reipold EI, Farahat A, Elbeeh A, Soliman R, Aza EB, Jamil MS, Johnson CC, Shiha G, Easterbrook P. Usability and acceptability of self-testing for hepatitis C virus infection among the general population in the nile delta region of Egypt. BMC Public Health. 2021;21(1):1188. 34158006 10.1186/s12889-021-11169-x PMC8218412

[18] Perazzo H, Castro R, Villela-Nogueira C, Torres M, Silva SL, Cardoso SW, Grinsztejn B, Veloso VG. Acceptability and usability of oral fluid HCV self-testing for hepatitis C diagnosis: A systematic review and meta-analysis. J Viral Hepat. 2023;30(11):838–47. 37485619 10.1111/jvh.13876

[19] Duah E, Ephraim RKD, Mathebula EM, Mashamba-Thompson TP. REASSURED evaluation of the bioline HCV point-of-care testing for diagnosing hepatitis C virus infection in primary healthcare settings of Ghana: a study protocol. BMJ Open. 2024;14(11):e082416. 39521466 10.1136/bmjopen-2023-082416 PMC12083280

[20] Piggott T, Moja L, Garcia CAC, Akl EA, Banzi R, Huttner B, Kredo T, Lavis JN, Schünemann HJ. User-experience testing of an evidence-to-decision framework for selecting essential medicines. PLOS Global Public Health. 2024;4(1):e0002723. 38206901 10.1371/journal.pgph.0002723 PMC10783770

[21] Uakarn C, Chaokromthong K, Sintao N. Sample size Estimation using Yamane and Cochran and Krejcie and Morgan and green formulas and Cohen statistical power analysis by G*Power and comparisons. Apheit Int J. 2021;10(2):76–88.

[22] Lewis JR. The system usability scale: past, present, and future. Int J Human–Computer Interact. 2018;34(7):577–90.

[23] Heerink JS, Gemen E, Oudega R, Hopstaken R, Geersing GJ, Kusters R. Analytical performance and user-friendliness of five novel point-of-care D-dimer assays. Scand J Clin Lab Investig. 2020;80(5):433–40. 32459511 10.1080/00365513.2020.1768586

[24] StataCorp. Stata statistical software. College Station, TX: StataCorp LLC.; 2019.

[25] ATLAS.ti. ATLAS.ti Desktop & Web| Free Trial| Get started for free!. ATLAS.ti. 2024. [cited 2024 May 18]. Available from: https://atlasti.com/free-trial-version.

[26] Maguire M, Delahunt B. Doing a thematic analysis: A practical, Step-by-Step guide for learning and teaching scholars. AISHE-J. 2017;8(3):3351–33514.

[27] O’Connor C, Joffe H. Inter-coder reliability in qualitative research: debates and practical guidelines. Int J Qualitative Methods. 2020;19:1609406919899220.

[28] Krüger LJ, Lindner AK, Gaeddert M, Tobian F, Klein J, Steinke S, Lainati F, Schnitzler P, Nikolai O, Mockenhaupt FP, Seybold J, Corman VM, Jones TC, Pollock NR, Knorr B, Welker A, Weber S, Sethurarnan N, Swaminathan J, Solomon H, Padmanaban A, Thirunarayan M, de Vos LP, Ongarello M, Sacks S, Escadafal JA, Denkinger C. A multicenter clinical diagnostic accuracy study of surestatus, an affordable, WHO emergency Use-Listed, rapid, Point-Of-Care Antigen-Detecting diagnostic test for SARS-CoV-2. Microbiol Spectr. 2022;10(5):e01229–22. 36066256 10.1128/spectrum.01229-22 PMC9604065

[29] Majam M, Fischer A, Ivanova Reipold E, Rhagnath N, Msolomba V, Lalla-Edward ST. A Lay-User assessment of hepatitis C virus Self-Testing device usability and interpretation in Johannesburg, South Africa. Diagnostics. 2021;11(3):463. 33800060 10.3390/diagnostics11030463 PMC8000311

[30] Solomon SS, Mehta SH, Srikrishnan AK, Solomon S, McFall AM, Laeyendecker O, Celentano DD, Iqbal SH, Anand S, Vasudevan CK, Saravanan S, Lucas GM, Kumar MS, Sulkowski MS, Quinn TC. Burden of hepatitis C virus disease and access to hepatitis C virus services in people who inject drugs in India: a cross-sectional study. Lancet Infect Dis. 2015;15(1):36–45. 2014/12/03. 25486851 10.1016/S1473-3099(14)71045-X PMC4503257

