# Supplementary material for: Assessing user experience with the Bioline™ HCV point-of-care test in primary healthcare settings: a mixed-methods study
Source: BMC Health Serv Res. 2025 Apr 1;25:484. doi: 10.1186/s12913-025-12634-8 (PMC11963430; doi:10.1186/s12913-025-12634-8)
Supplement: Supplementary file 1 — Additional file 1. [file 12913_2025_12634_MOESM1_ESM.docx]

**Additional file 1**

**PHC clinics included and corresponding study participants**

| **District** | **CHPS** | **Available staff** | **Study participants** |
| --- | --- | --- | --- |
| **Cape Coast** | |  |  |
| Adisadel | Ankaful CHPS | 3 | 3 |
|  | Brimso Sanford CHPS | 3 | 3 |
|  | Essuakyir CHPS | 3 | 2 |
|  | Krofufrodu CHPS | 3 | 3 |
|  | Mpeasem CHPS | 4 | 2 |
|  | Kakumdo CHPS | 4 | 4 |
| Cape Coast Central | Siwdu CHPS |  |  |
| Efutu | Dehia CHPS | 3 | 1 |
|  | Efutu Mampong CHPS | 3 | 2 |
| Ewim | Nkanfoa CHPS | 3 | 3 |
| UCC | Akotokyir CHPS | 3 | 3 |
|  | Amamoma CHPS | 2 | 1 |
|  | Duakor/Abakam CHPS | 2 | 1 |
| **Komenda-Edina-Eguafo-Abirem District** | |  |  |
| Abrem Agona | Abrem Essiam CHPS | 2 | 1 |
|  | Benyadze CHPS | 2 | 1 |
|  | Berase CHPS | 3 | 1 |
|  | Eguafo CHPS | 3 | 3 |
|  | Egyeikrom Refugee Camp CHPS | 3 | 3 |
| Elmina | Ampenyi CHPS | 3 | 1 |
|  | Ankwanda CHPS | 2 | 1 |
|  | Brenu Akyinim CHPS | 1 | 1 |
| Kissi | Abeyee CHPS | 3 | 2 |
|  | Antseambua CHPS | 2 | 1 |
|  | Besease CHPS | 2 | 1 |
|  | Kokoado CHPS | 1 | 1 |
| Komenda | Aburansa CHPS | 3 | 3 |
|  | Kafodzidzi CHPS | 4 | 4 |
|  | Nsadwir CHPS | 2 | 2 |
| **Mfantseman Districts** | |  |  |
| Anomabo | Nsanfo CHPS | 2 | 2 |
|  | Taido CHPS | 2 | 1 |
| Biriwa | Akatakyiwa CHPS | 2 | 2 |
|  | Asafora CHPS | 3 | 3 |
|  | Eguase CHPS | 2 | 2 |
|  | Kobina Ansah CHPS | 1 | 1 |
| Dominase | Akobima CHPS | 1 | 1 |
|  | Duadze CHPS | 1 | 1 |
|  | Kyeakor CHPS | 2 | 2 |
|  | Taabosom CHPS | 1 | 1 |
| Mankessim | Mankessim Market CHPS | 6 | 6 |
| Saltpond | Ankaful CHPS | 3 | 3 |
|  | Hini CHPS | 1 | 1 |
|  | Kuntu CHPS | 1 | 1 |
| Total | 42 | 100 | 81 |
