# Supplementary material for: Assessing user experience with the Bioline™ HCV point-of-care test in primary healthcare settings: a mixed-methods study
Source: BMC Health Serv Res. 2025 Apr 1;25:484. doi: 10.1186/s12913-025-12634-8 (PMC11963430; doi:10.1186/s12913-025-12634-8)

# A. The specimen dropper, assay diluent, alcohol swab and lancet. B. Test device in foil pouch. C. Test device removed from foil pouch. D. Sample droppers. E. HCW collecting blood sample by finger prick. F. HCW collecting blood sample with the sample dropper.


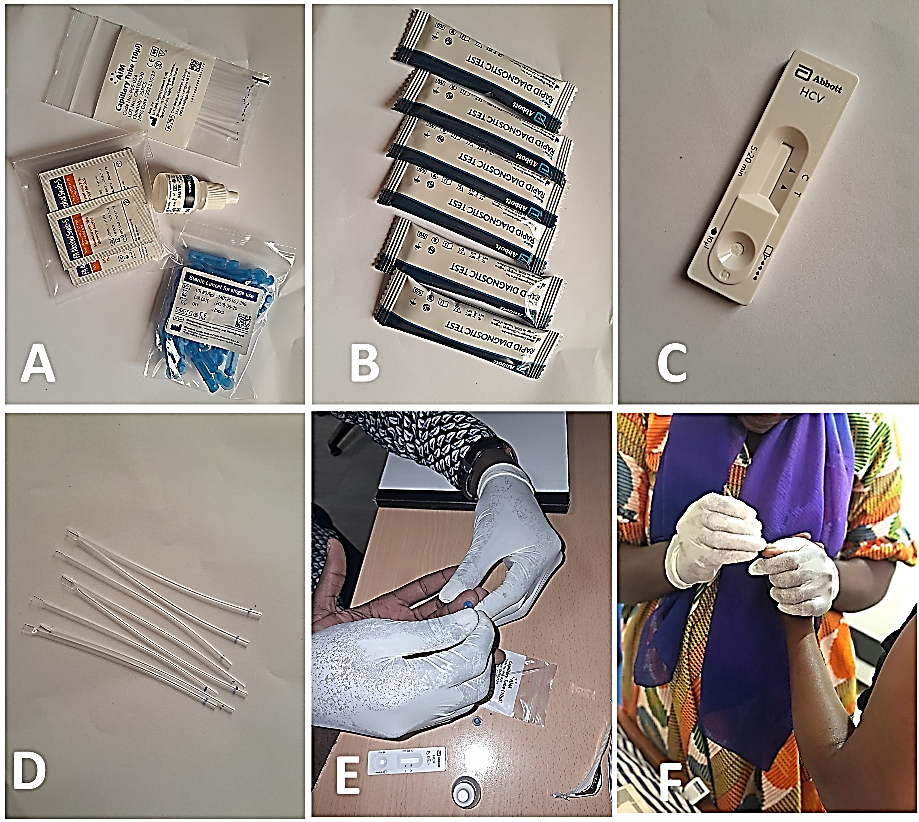

Supplement: Supplementary file 2 — Additional file 2. [file 12913_2025_12634_MOESM2_ESM.docx]
