# Supplementary material for: Assessing user experience with the Bioline™ HCV point-of-care test in primary healthcare settings: a mixed-methods study
Source: BMC Health Serv Res. 2025 Apr 1;25:484. doi: 10.1186/s12913-025-12634-8 (PMC11963430; doi:10.1186/s12913-025-12634-8)
Supplement: Supplementary file 3 — Additional file 3. [file 12913_2025_12634_MOESM3_ESM.docx]

**Additional file 3**

**Questionnaire**

| **Unique identifier** |  |
| --- | --- |
| **Name of PHC clinic** |  |
| **Name of District** |  |
| **Name of sub-district** |  |
| **Name of Community** |  |
| **Condition of road network** | Non-pliable road [ ] Poor road but pliable [ ] Good road [ ] |
| **Availability of air-conditioned storage room** | Yes [ ] No [ ] |
| **Date** |  |

| 1. **Socio-demographic information** |
| --- |
| Age (years): |
| Sex: Male [ ] Female [ ] |
| Level of education: Certificate [ ] Diploma [ ] Degree [ ] Masters [ ] Ph.D. [ ] |
| Occupation/ profession: |
| Professional rank/cadre: |
| Total years of working experience: |
| Years of working in this clinic: |
| Leadership position (if any): |

| 1. **Audit 1 (hands-on)** | **Yes** | **No** | **N/A** | **Comment** |
| --- | --- | --- | --- | --- |
| 1. Did the study participant wash hands and dried before starting to test? |  |  |  |  |
| 1. Did the study participant read/use the information sheet or test insert? |  |  |  |  |
| 1. Was it difficult for the study participant to remove the test device from the foil pouch? |  |  |  |  |
| 1. Did the study participant successfully place material on a flat surface and open all pouches? |  |  |  |  |
| 1. Did the study participant wear examination gloves? |  |  |  |  |
| 1. Did the study participant label the test kit? |  |  |  |  |
| 1. Did the study participant correctly choose the ring or middle finger? |  |  |  |  |
| 1. Did the study participant massage and warm their hands? |  |  |  |  |
| 1. Did the study participant clean the finger with an alcohol swab and let it dry? |  |  |  |  |
| 1. Did the study participant successfully press down firmly to prick their skin? |  |  |  |  |
| 1. Did the study participant safely discard the lancet? |  |  |  |  |
| 1. Did the study participant successfully wipe away the first drop of blood with tissue and then rub it to create a second large drop of blood? |  |  |  |  |
| 1. Did the study participant use the specimen dropper to collect the drop of blood? |  |  |  |  |
| 1. Did the study participant collect the blood up to the marked ring of the specimen dropper (10µl)? |  |  |  |  |
| 1. Did the study participant successfully dispense the whole blood into the round specimen well (marked ‘S’) of the device? |  |  |  |  |
| 1. Did the study participant safely discard the used specimen dropper? |  |  |  |  |
| 1. Was the study participant able to easily turn the cap to open the assay diluent? |  |  |  |  |
| 1. Did the study participant dispense exactly 4 drops of the assay diluent into the same round well of the device? |  |  |  |  |
| 1. Did the study participant close the cap of the assay diluent after use? |  |  |  |  |
| 1. Did the study participant read the test results within the stipulated time (5-20 mins)? |  |  |  |  |
| 1. Did the study participant interpret the results correctly? |  |  |  |  |
| 1. Did the study participant safely discard the used test kit? |  |  |  |  |
| 1. If invalid results were obtained, did the study participant attempt to retest? |  |  |  |  |

| 1. **Audit 2 (User-perception)** | **Very easy** | **Easy** | **Not easy** |
| --- | --- | --- | --- |
| 1. Reading/using the information sheet or test insert |  |  |  |
| 1. Removing the test device from the foil pouch |  |  |  |
| 1. Sample collection using finger prick |  |  |  |
| 1. Collecting the drop of blood with the specimen dropper |  |  |  |
| 1. Dispensing the whole blood into the round specimen well (marked ‘S’) of the device |  |  |  |
| 1. Opening the cap of the assay diluent |  |  |  |
| 1. Dispensing the assay diluent into the same well of the device? |  |  |  |
| 1. Timing the test |  |  |  |
| 1. Reading the test results |  |  |  |
| 1. Interpreting the test results |  |  |  |

| 1. **The System Usability Scale (SUS)-Quantitative** | **Strongly Disagree** | **Disagree** | **Neutral** | **Agree** | **Strongly Agree** |
| --- | --- | --- | --- | --- | --- |
|  | **1** | **2** | **3** | **4** | **5** |
| 1. I think that I would like to use this test frequently. |  |  |  |  |  |
| 1. I found the test unnecessarily complex. |  |  |  |  |  |
| 1. I thought the test kit was easy to use. |  |  |  |  |  |
| 1. I think that I would need the support of a technical person to be able to use this test. |  |  |  |  |  |
| 1. I found the various functions in this test were well integrated. |  |  |  |  |  |
| 1. I thought there was too much inconsistency in this test. |  |  |  |  |  |
| 1. I would imagine that most people would learn to use this test very quickly. |  |  |  |  |  |
| 1. I found the test very awkward to use. |  |  |  |  |  |
| 1. I felt very confident using the test. |  |  |  |  |  |
| 1. I needed to learn a lot of things before I could get going with this test. |  |  |  |  |  |

| 1. **Inter-reader concordance (Test on each other)** | | |
| --- | --- | --- |
| **Participant's HCV POC test result** | **Researcher’s HCV POC test results** | **Comment** |
|  |  |  |
|  |  |  |
| **Inter-operator concordance** | | |
| **Participant’s HCV POC test result** | **Researcher’s HCV POC test results** | **Comment** |
|  |  |  |
|  |  |  |

| 1. **Inter-reader concordance (Random standard blood sample)** | | |
| --- | --- | --- |
| **Participant’s HCV POC test result** | **Researcher’s HCV POC test results** | **Comment** |
|  |  |  |
|  |  |  |
| **Inter-operator concordance** | | |
| **Participant’s HCV POC test result** | **Researcher’s HCV POC test results** | **Comment** |
|  |  |  |
|  |  |  |

**Thank you for taking the time to respond to this questionnaire.**
