# Supplementary material for: Assessing user experience with the Bioline™ HCV point-of-care test in primary healthcare settings: a mixed-methods study
Source: BMC Health Serv Res. 2025 Apr 1;25:484. doi: 10.1186/s12913-025-12634-8 (PMC11963430; doi:10.1186/s12913-025-12634-8)
Supplement: Supplementary file 4 — Additional file 4. [file 12913_2025_12634_MOESM4_ESM.docx]

**Additional file 4**

| **A. User-perception (Interview guide)-Qualitative** |
| --- |
| 1. Please share your general experience of using the Bioline™ HCV test kit |
| 2. How was the instructions sheet useful or not to you? Probe. |
| 3. If any, what difficulty or challenges did you face while using the Bioline™ HCV test kit? |
| 4. Please share your experience of the sample collection procedure for the HCV test using the Bioline™ HCV test kit? |
| 5. What steps did you take to ensure the reliability of the test results? |
| 6. How does the stipulated time for reading the results match or differ from the time on the test instructions? |
| 7. How confident were you when performing the test? Rate your confidence as  “very confident”, “confident” and “not confident” and explain |
| 8. In your experience, how do you think the simplicity of the test in terms of equipment required for the Bioline™ HCV test makes it easier to use? |
| 9. How does the simplicity of the Bioline™ HCV test affect its usability, especially in terms of storage infrastructure or space available in your facility? |
| 10. What challenges are you likely to face with storage of the test kit? |
| 11. What factors will you and your facility consider before using the Bioline™ HCV test in your facility? |
| 12. Are you willing to use this test in this facility and in future? Explain |
| 13. Would you recommend this test for use in other PHC clinics? Explain |
| 14. What feature about this test would you recommend to be changed by the manufacturer? |
| 15. What feature about the test would you prefer to be added in subsequent models by the manufacturer? |
