# Supplementary material for: Assessing user experience with the Bioline™ HCV point-of-care test in primary healthcare settings: a mixed-methods study
Source: BMC Health Serv Res. 2025 Apr 1;25:484. doi: 10.1186/s12913-025-12634-8 (PMC11963430; doi:10.1186/s12913-025-12634-8)
Supplement: Supplementary file 6 — Additional file 6. [file 12913_2025_12634_MOESM6_ESM.docx]

**Additional file 6**

**Characteristics of study participants included in the in-depth interviews**

| S/N | District | Age | Sex | Level of  education | Occupation/  Profession | Rank | Total years of working experience | Years of working in this PHC clinic | Leadership role |
| --- | --- | --- | --- | --- | --- | --- | --- | --- | --- |
| 1 | Mfantseman | 37 | Female | Diploma | Midwife | Senior Staff Midwife | 10 | 2 | In-charge |
| 2 | Mfantseman | 43 | Female | Certificate | Nurse | Senior Enrolled Nurse | 8 | 3 | In-charge |
| 3 | Mfantseman | 30 | Female | Certificate | Nurse | Senior Community Health Nurse | 5 | 5 | None |
| 4 | Mfantseman | 43 | Female | Diploma | Midwife | Senior Midwifery Officer | 20 | 4 | In-charge |
| 5 | KEEA | 33 | Female | Certificate | Nurse | Senior Community Health Nurse | 6 | 6 | In-charge |
| 6 | KEEA | 33 | Female | Degree | Midwife | Senior Staff Midwife | 11 | 1 | In-charge |
| 7 | Mfantseman | 34 | Female | Certificate | Nurse | Senior Community Health Nurse | 5 | 5 | None |
| 8 | Cape Coast | 34 | Female | Certificate | Nurse | Principal Community Health Nurse | 11 | 7 | In-charge |
| 9 | KEEA | 34 | Female | Certificate | Nurse | Senior Community Health Nurse | 5 | 2 | In-charge |
| 10 | Mfantseman | 34 | Female | Degree | Midwife | Midwifery Officer | 16 | 2 | In-charge |
| 11 | KEEA | 37 | Female | Diploma | Midwife | Midwifery Officer | 13 | 1 | In-charge |
| 12 | KEEA | 29 | Female | Certificate | Nurse | Senior Community Health Nurse | 5 | 1 | In-charge |
| 13 | Mfantseman | 38 | Female | Certificate | Nurse | Principal Community Health Nurse | 12 | 4 | In-charge |
| 14 | Mfantseman | 33 | Male | Degree | Nurse | Nursing Officer | 12 | 1 | In-charge |
| 15 | Mfantseman | 34 | Female | Certificate | Nurse | Principal Community Health Nurse | 10 | 5 | In-charge |
| 16 | Mfantseman | 30 | Female | Certificate | Nurse | Senior Community Health Nurse | 4 | 1 | None |
| 17 | Mfantseman | 37 | Female | Degree | Midwife | Midwifery Officer | 7 | 5 | In-charge |
| 18 | Mfantseman | 39 | Female | Certificate | Nurse | Community Health Nurse | 14 | 6 | None |
| 19 | Cape Coast | 43 | Female | Diploma | Midwife | Senior Midwifery Officer | 20 | 1 | In-charge |
| 20 | Mfantseman | 34 | Female | Diploma | Midwife | Midwifery Officer | 9 | 2 | In-charge |
| 21 | Mfantseman | 36 | Male | Certificate | Nurse | Senior Community Health Nurse | 7 | 5 | In-charge |
| 22 | Mfantseman | 36 | Female | Certificate | Nurse | Principal Community Health Nurse | 9 | 4 | None |
