# Supplementary material for: Assessing user experience with the Bioline™ HCV point-of-care test in primary healthcare settings: a mixed-methods study
Source: BMC Health Serv Res. 2025 Apr 1;25:484. doi: 10.1186/s12913-025-12634-8 (PMC11963430; doi:10.1186/s12913-025-12634-8)
Supplement: Supplementary file 7 — Additional file 7. [file 12913_2025_12634_MOESM7_ESM.docx]

**Additional file 7**

**Emerging themes, sub-themes and quotes**

| **SN** | **Themes** | **Sub-themes** | **Quotes** |
| --- | --- | --- | --- |
| **1** | User experiences | Ease-of-use of the test | *“Every equipment needed for the test is found in the small box. You don’t need any other equipment to perform the test, which makes it very simple to use”* (HCW of PHC clinic 18)  *“The test kit is quite compact and lightweight. It’s something we can easily work with”* (HCW of PHC clinic 11) |
|  |  | Rapidity of the test | *“It is a quick way of determining an individual’s HCV status. Within 10 to 20 minutes, you can know if a patient has hepatitis C or not”* (HCW of PHC clinic 2) |
|  |  | Familiarity of test procedures | *“Although this is a hepatitis C test, it is not new to us. As a CHPS compound, we are familiar with performing malaria RDTs since about 99% of our clients are malaria cases. We do malaria RDTs daily before prescribing malaria medications. Using this test felt like part of our normal routine”* (HCW of PHC clinic 6) |
|  |  | Experience with the test instruction sheet | *“The leaflet is easy to understand. It includes pictures illustrating the various procedures, such as how to prick the finger, collect the blood, and drop the buffer. It provides both text and picture explanations”* (HCW of PHC clinic 9) |
|  |  | Experience with sample collection procedure | *“It was very easy. Patients should be okay with the finger prick”* (HCW of PHC clinic 6) |
| **2** | Storage requirements | Space-efficient design of the test | *“No, we don’t need any larger space to store this. It can be stored with the other drugs in our facility”* (HCW of PHC clinic 7) |
|  |  | Adaptability to existing storage conditions | *“There won’t be any challenges. Although we don’t have an air-conditioned room, we have a ceiling fan. Since our drugs are stored under the same conditions and are okay, the test kits will be equally well-preserved”* (HCW of PHC clinic 6) |
| **3** | Test acceptability | Willingness to use test in the PHC clinics | “*Yes. As the first point of contact in the hierarchy of Ghana’s health service, having this test kit will make our work much easier. Clients will no longer need to travel long distances and incur financial costs for laboratory tests”* (HCW of PHC clinic 7) |
|  |  | Willingness to recommend to other PHC clinics | *“Yes. Most people in these communities see hospitals as something big, so they are scared to go there. They are comfortable coming to the CHPS compounds since we are closer to their homes. It will also make the HCV diagnostic service easily accessible to them”* (HCW of PHC clinic 15)  *“Yes, it will be very helpful because most community members prefer accessing healthcare in CHPS zones (PHC clinics). Even when referred to a higher level of care, they often refuse to go. They are scared of larger health facilities. Having this test will help us diagnose them here and provide appropriate follow-up treatment”* (HCW of PHC clinic 12) |
| **4** | Challenges encountered | Challenge with opening the test foil | *“I faced a challenge with opening the pouch containing the cassette. The malaria RDT we are used to has a little cut at the edge that makes opening much easier, unlike this one”* (HCW of PHC clinic 19) |
|  |  | Challenge with reading/using the test instruction sheet | *“When I saw the leaflet, I got scared. I was like, “Ei, what is this big thing? When will I even finish reading this? (She laughed)”* (HCW of PHC clinic 19**)**  *“I had a little difficulty with the pamphlet (instruction sheet). It’s quite large and includes multiple languages, which made it hard to locate the specific information needed to perform the test”* (HCW of PHC clinic 11)  “*The font is too small for some of us with poor eyesight”* (HCW of PHC clinic 13) |
|  |  | Challenge with using the specimen dropper | *“I had a problem with the pipette. I had to press it before picking up the sample. It requires some skill to do that. If you don’t know how, you can’t take enough sample for the test”* (HCW of PHC clinic 14) |
|  |  | Challenge with timing | *“The timing is okay, but if you’re not careful, you might miss it. You could forget to time properly”* (HCW of PHC clinic 1) |
| **5** | Suggestions for improvement | Suggestion to modify the test package | *“Okay. I would suggest they improve the packaging. If the pipette (dropper) and lancet are included in the foil with the cassette, it would be more convenient. That way, when you pick one, everything you need is inside. Currently, since the pipettes and lancets are packed separately, sometimes we run out of them even though we still have cassettes available. Including them together would solve this issue”* (HCW of PHC clinic 1) |
|  |  | Suggestion to include additional consumables | *“They should include additional droppers since we may accidentally drop them while working”* (HCW of PHC clinic 2)  ***“****Adding lancet holders would be very helpful, as it would reduce clients’ fear of needles by hiding the needle during finger pricks…* (HCW of PHC clinic 3)  *“You should add a lancet holder to automate the pricking process and protect us from needle pricks during our work. This would also reduce the pain caused by the prick”* (HCW of PHC clinic 4)  *“We often lack sufficient gloves. Including gloves, even simple rubber gloves, in the test package would greatly enhance safety”* (HCW of PHC clinic 7)  *“…including a timer would be beneficial to help with timing, as we might forget to check the result on time”* (HCW of PHC clinic 3) |
|  |  | Suggestion to modify specimen dropper | *“I would recommend changing the pipette (dropper). It’s difficult to pick the sample with it compared to what we are used to with the malaria RDT. With the malaria RDT, you don’t need to press or hold anything to pick the sample. It picks the sample with just a touch”* (HCW of PHC clinic 6) |
|  |  | Suggestion to modify test instruction sheet | *“…the leaflet should be simplified. I don’t know if this is in Chinese, but if you’re bringing the test to this level of healthcare in Ghana, most of us are certificate and diploma holders. When someone sees a language other than English, they might not even continue to look at the instructions. So, I would recommend designing a country-specific leaflet with test instructions in diagrams showing the steps, to make it simpler and easier to read”* (HCW of PHC clinic 14) |
