# Supplementary figures and images for: Assessing user experience with the Bioline™ HCV point-of-care test in primary healthcare settings: a mixed-methods study
Source: BMC Health Serv Res. 2025 Apr 1;25:484. doi: 10.1186/s12913-025-12634-8 (PMC11963430; doi:10.1186/s12913-025-12634-8)

# **A. Challenge with the test instruction sheet B. Challenge with the sample dropper**

**
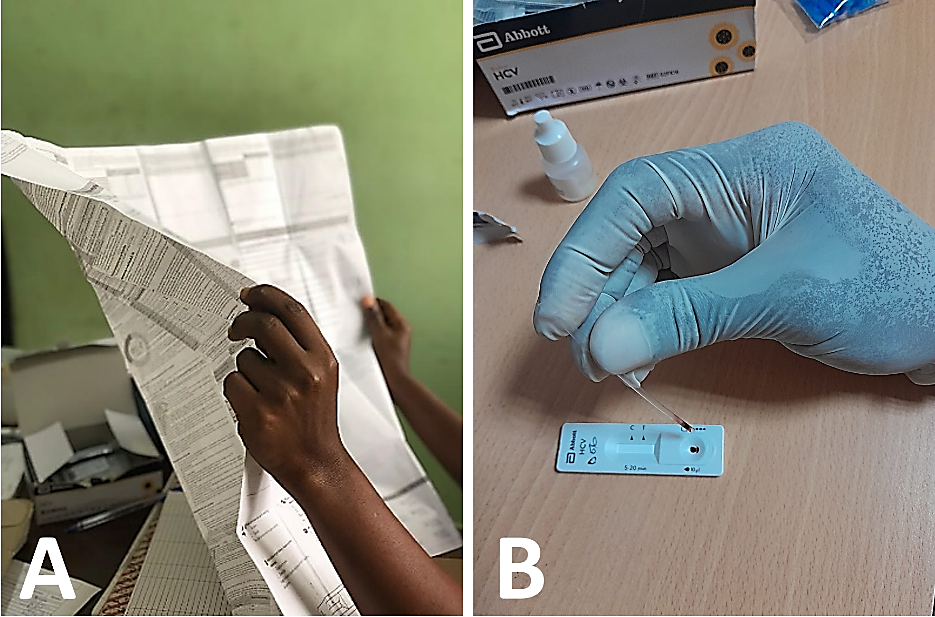
**

Supplement: Supplementary file 8 — Additional file 8. [file 12913_2025_12634_MOESM8_ESM.docx]
