# Supplementary material for: Assessing user experience with the Bioline™ HCV point-of-care test in primary healthcare settings: a mixed-methods study
Source: BMC Health Serv Res. 2025 Apr 1;25:484. doi: 10.1186/s12913-025-12634-8 (PMC11963430; doi:10.1186/s12913-025-12634-8)
Supplement: Supplementary file 9 — Additional file 9. [file 12913_2025_12634_MOESM9_ESM.docx]

Inter-rater concordance: person-testing and testing with standard sample

| **Inter-reader concordance** | | | | | **Inter-operator concordance** | | | | |
| --- | --- | --- | --- | --- | --- | --- | --- | --- | --- |
| **Person-testing** | Re-reading by research assistant | | | | **Person-testing** | Re-testing by research assistant | | | |
| HCW | Negative | Positive | Invalid | Total | HCW | Negative | Positive | Invalid | Total |
| Negative | 81 | 0 | 0 | 81 | Negative | 81 | 0 | 0 | 81 |
| Positive | 0 | 0 | 0 | 0 | Positive | 0 | 0 | 0 | 0 |
| Invalid | 0 | 0 | 0 | 0 | Invalid | 0 | 0 | 0 | 0 |
| Total | 81 | 0 | 0 | 81 | Total | 81 | 0 | 0 | 81 |
| Invalid rating: 0% | | | | | Invalid rating: 0% | | | | |
| Concordance: 100% | | | | | Concordance: 100% | | | | |
| **Standard sample** | Re-reading by research assistant | | | | **Standard sample** | Re-testing by research assistant | | | |
| HCW* | Negative | Positive | Invalid | Total | HCW | Negative | Positive | Invalid | Total |
| Negative | 26 | 0 | 0 | 26 | Negative | 26 | 0 | 0 | 26 |
| Positive | 0 | 55 | 0 | 55 | Positive | 0 | 55 | 0 | 55 |
| Invalid | 0 | 0 | 0 | 0 | Invalid | 0 | 0 | 0 | 0 |
| Total | 26 | 55 | 0 | 81 | Total | 26 | 55 | 0 | 81 |
| Invalid rating: 0% | | | | | Invalid rating: 0% | | | | |
| Concordance: 100%, Cohen’s kappa coefficient:1.00±0.11, p-value<0.001 | | | | | Concordance: 100%, Cohen’s kappa coefficient:1.00±0.11, p-value<0.001 | | | | |

*HCW: Healthcare worker
